# Perioperative Dexmedetomidine for outpatient cataract surgery: a systematic review

**DOI:** 10.1186/s12871-020-00973-4

**Published:** 2020-04-04

**Authors:** James Harvey Jones, Robin Aldwinckle

**Affiliations:** UC Davis Department of Anesthesiology and Pain Medicine, University of California Davis Medical Center, 4150 V Street, PSSB Suite 1200, Sacramento, CA 95817 USA

**Keywords:** Dexmedetomidine, Outpatient surgery, Ambulatory surgery, Cataract surgery

## Abstract

**Background:**

Cataract surgery is one of the most common procedures performed worldwide in the elderly. Various medications can provide effective anesthesia and analgesia for cataract surgery, but undesirable side effects limit the utility of each medication or combination of medications. Dexmedetomidine may serve as an anesthesia adjunct for outpatient cataract surgery in the elderly.

**Methods:**

Searches were conducted in Cochrane, Embase, and PubMed for randomized clinical trials investigating the use of dexmedetomidine in adult patients undergoing outpatient, or ambulatory, cataract surgery with sedation and topical or peribulbar block. Ninety-nine publications were identified, of which 15 trials satisfied the inclusion criteria. A total of 914 patients were included in this review. The following data were collected: American Society of Anesthesiologists’ (ASA) physical status and age of study patients; method of blinding and randomization; medication doses and routes of administration; and intraoperative levels of sedation. We also recorded statistically significant differences between dexmedetomidine and other study medications or placebo with respect to the following outcomes: hemodynamic and respiratory parameters; pain; sedation; post-operative nausea and vomiting (PONV); discharge from post-anesthesia care unit (PACU) or recovery times; patient satisfaction; surgeon satisfaction; and effects on intraocular pressure (IOP).

**Results:**

Hypotension with or without bradycardia was reported following bolus doses of dexmedetomidine ranging from 0.5–1.0 mcg/kg with or without a continuous dexmedetomidine infusion. Delayed PACU discharge times were associated with the use of dexmedetomidine, but no clear association was identified between delayed recovery and higher levels of intraoperative sedation. Better analgesia and higher patient satisfaction were commonly reported with dexmedetomidine as well as reductions in IOP.

**Conclusions:**

Overall, this review demonstrates better analgesia, higher patient satisfaction, and reduced IOP with dexmedetomidine for outpatient cataract surgery when compared to traditional sedatives, hypnotics, and opioids. These benefits of dexmedetomidine, however, must be weighed against relative cardiovascular depression and delayed PACU discharge or recovery times. Therefore, the utility of dexmedetomidine for outpatient cataract surgery should be considered on a patient-by-patient basis.

## Background

Cataract is a leading cause of visual impairment that currently affects almost 25 million Americans and is projected to reach almost 50 million by 2050 [1]. Approximately 2 million cataract operations are performed in the United States each year, and the majority are done in elderly patients [2, 3].

Anesthesia for cataract surgery often includes sedatives and hypnotics (such as propofol, ketamine, or midazolam) with or without opioids, along with topical analgesia or peribulbar block. Propofol and benzodiazepines may induce persistent sedation and respiratory depression, particularly when they are administered in combination with opioids in elderly patients [4]. Opioids may also lead to nausea, vomiting, and perioperative neurocognitive disorders [5].

Ambulatory, or outpatient, cataract surgery demands quick resolution of anesthesia-related effects prior to patient discharge. Postoperative pain, sedation, nausea, vomiting, as well as hemodynamic and respiratory parameters that have not returned to an acceptable percentage of baseline may delay discharge, require additional monitoring, or lead to unexpected hospital admission.

Dexmedetomidine is an alpha-2 agonist with a half-life of approximately 2 h that provides sedation and analgesia without compromising oxygenation and ventilation [6, 7]. However, reported side effects of bradycardia, hypotension, hypertension, and nausea may restrict the use of dexmedetomidine in the ambulatory surgery setting [6–8]. Despite the popularity of cataract surgery and the widespread use of dexmedetomidine in anesthesia, no systematic review is currently available that assembles the evidence regarding dexmedetomidine for outpatient cataract surgery.

This systematic review attempts to synthesize the results from randomized clinical trials investigating the use of dexmedetomidine for sedation in adult patients undergoing outpatient cataract surgery. Our review focuses on patient and surgeon satisfaction as well as factors that impact perioperative outcomes: hemodynamic and respiratory parameters; pain; sedation; postoperative nausea and vomiting (PONV); post-anesthesia care unit (PACU) discharge or recovery times; and intraocular pressure (IOP).

## Methods

### Literature search

The authors (JHJ and RA) independently performed initial literature searches in PubMed, Embase, and Cochrane databases for articles published before January 1, 2019, using the following key terms in the English language: “dexmedetomidine” and “cataract surgery.” Additionally, the bibliographies of recovered publications were screened for relevant titles, which were then also reviewed. Eligible studies included randomized clinical trials of adult patients undergoing outpatient, or ambulatory, cataract surgery with sedation supplemented by topical analgesia or peribulbar block.

### Data collection

The following data were collected from each randomized clinical trial: age and American Society of Anesthesiologists’ (ASA) physical status of study patients; method of blinding and randomization; medication doses and routes of administration; and intraoperative level of sedation. We also recorded statistically significant differences between dexmedetomidine and other study medications or placebo with respect to the following outcomes: hemodynamic and respiratory parameters; pain; sedation; PONV; discharge from PACU or recovery times; patient satisfaction; surgeon satisfaction; and IOP. The methods by which the studies measured each outcome (such as Visual Analog Score, Numeric Rating Scale, Aldrete’s scoring system, and Ramsay Sedation Scale) were also noted. Data regarding outcomes not expected to impact patient discharge from an ambulatory surgery center (such as incidence of dry mouth) were not recorded.

### Definitions

Ambulatory surgery was defined as surgery after which the patients were discharged from the PACU to home and was considered synonymous with outpatient surgery for the purposes of this review. Unless the authors specifically stated otherwise, it was assumed that all patients were discharged from the PACU to home. Adverse events were only recorded if they were experienced by dexmedetomidine-treated patients. Adverse events of other study medications were not recorded given the heterogeneity of medications analyzed, and the focus of this review was on the effects of dexmedetomidine.

### Assessment of study quality

The quality of each study was assessed with 3 measures and given a score between 0 and 5 [9]. One point was awarded if the study was randomized and another point was awarded if the study utilized a double-blind approach. Additional points were given based on the appropriateness of randomization and blinding and if the study included a clear description of withdrawals or dropouts (or specifically stated that there were none). Points were not awarded for inappropriate randomization or blinding.

## Results

### Study selection

**Fig. 1:**
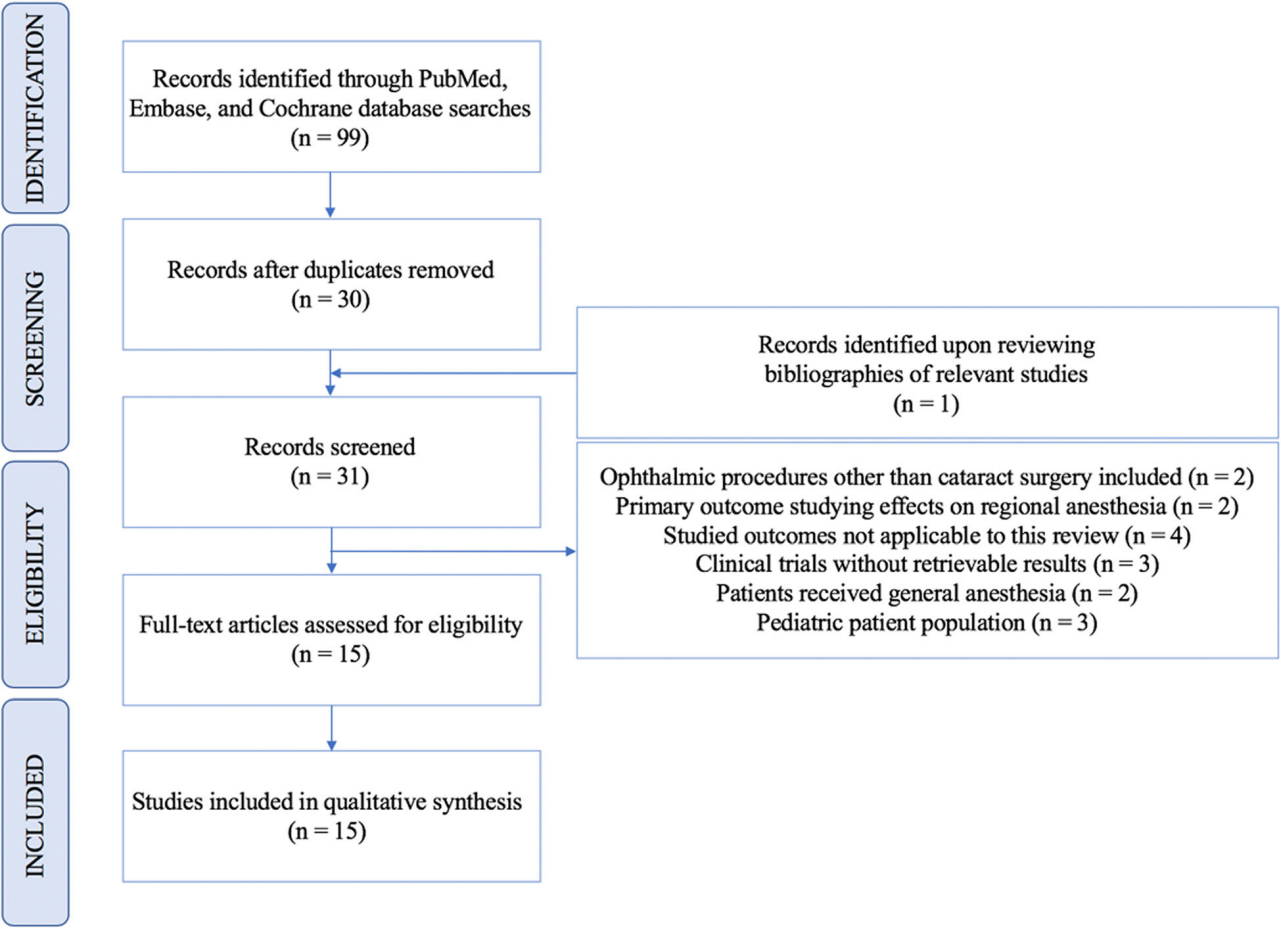
Identification and Screening of Publications

Literature searches in PubMed, Cochrane, and Embase databases identified 99 articles published prior to January 1, 2019. One additional publication was identified after reviewing the bibliographies of recovered studies. After removing duplicates from database searches, 31 publications were screened and 16 were excluded due to the following reasons: ophthalmic procedures other than cataract surgery included in the study (number (n) = 2), primary outcome regarding the effects of dexmedetomidine on regional anesthesia for ophthalmic surgery (*n* = 2), outcomes not applicable to this review (*n* = 4), clinical trials without retrievable results (*n* = 3), study patients received general anesthesia (*n* = 2), and pediatric patient population (*n* = 3). All of the remaining 15 eligible studies were included for qualitative synthesis. The method used to identify and screen publications is displayed in Fig. 1: Identification and Screening of Publications. Additional information regarding study designs and demographic information are shown in Table 1.

**Table 1:** Study Design, Quality Score, and Demographic Data of Included Studies

Study	Quality Score	N^a^	ASA^b^	Age in Dexmedetomidine Group(s)^c^		Age in Other Group(s)^c^	
Abdalla [23]	3	40	–	63 (11)		60 (13)	
Alhashemi [12]	4	44	I-III	61 (34–79)		61 (40–75)	
Altiparmak [24]	5	80	II-III	58 (5.7)		57 (5.8)	
Apan [11]	3	90	I-III	65.7 (11.3)		midazolam	65.8 (11.8)
						saline	66.3 (9.8)
Ayoglu [21]	1	40	I-II	50–75		50–75	
Dogan [13]	4	80	I-II	50–70		50–70	
Erdurmus [14]	3	44	–	67.41 (9.83)		69.46 (9.99)	
Ghodki [15]	4	60	I-II	62.6 (6.5)		61.4 (6.9)	
Kermany [10]	1	100	I-II	50.26 (9.44)		51.9 (10.2)	
Muttu [16]	1	40	–	Unknown		Unknown	
Na [17]	1	31	I-III	60.8 (11.4)		57.4 (13.4)	
Park [22]	0	80	I-III	60–80		60–80	
Virkilla [19]	0	35	I-III	0.25 mcg/kg^d^	69.0 (8.7)	60.8 (12.2)	
				0.5 mcg/kg	70.2 (3.3)		
				0.75 mcg/kg	67.8 (6.7)		
				1.0 mcg/kg	66.6 (15.0)		
				1.5 mcg/kg	71.4 (10.9)		
Virkilla [20]	4	90	I-III	63.3 (11.2)		midazolam	66.2 (9.5)
						placebo	66.6 (10.3)
Yagan [18]	3	60	I-III	68.6 (8.4)		66.4 (6.3)	

^a^*N* number of patients studied, ^b^*ASA* American Society of Anesthesiologists’ classification of Physical Health score, ^c^Data are mean (standard deviation or age range) or age range, ^d^*mcg/kg* micrograms per kilogram

### Quality of studies

Although each study in this review utilized a randomized design, the method of randomization was frequently inappropriate and led to decreased quality scores. Failure to describe withdrawals and dropouts also accounted for low quality scores as noted in Table 1. Most studies utilized a double-blind approach that was considered appropriate.

### Routes of administration, dosing, and titration of medications

Two studies titrated dexmedetomidine, remifentanil, and midazolam to pre-determined target levels of sedation as defined by Bispectral index (BIS) scores 70–80 and > 85 [10, 11]. Of the remaining studies, 7 titrated medications to Ramsay Sedation Scale (RSS) scores of 2 or 3 [12–18]. Two studies administered intramuscular (IM) dexmedetomidine 45 min prior to the regional block [19, 20]. One study employed patient-controlled sedation [21]. Another study utilized target effect site concentrations to titrate remifentanil [22]. Table 2 shows the medication doses, routes of administration, total medication administered, and intraoperative levels of sedation.

**Table 2:** Route of Administration, Doses, Titration Method, and Total Medication Administered of Included Studies

Study	Route^a^	Dexmedetomidine Bolus Dose(s)^b^	Bolus Duration (mins)^*c*^	Dexmedetomidine Infusion Rate^d^	Other Treatment Group(s)	Medication Titration	Total Medication Administered^e^		
Abdalla [23]	IV	0.5 mcg•kg^− 1^•hr.^− 1^	10	0.2 mcg•kg^− 1^•hr.^− 1^ over 50 mins	saline	NA	Unknown		
Alhashemi [12]	IV	1 mcg/kg	10	0.1–0.7 mcg•kg^− 1^•hr.^− 1^	midazolam 20 mcg/kg then 0.5 mg PRN^f^	RSS^g^ 3	dexmedetomidine 79.5 (21.7) mcg		
							midazolam 1.5 (0.6) mg		
Altiparmak [24]	IV	–	NA	0.4 mcg•kg^− 1^•hr.^− 1^	remifentanil 0.05 mcg•kg^− 1^•min^− 1^	Unknown	Unknown		
Apan [11]	IV	–	NA	0.25 mcg•kg^− 1^•hr.^− 1^	midazolam 25 mcg•kg^− 1^•hr.^− 1^ or saline	BIS^h^ > 85	dexmedetomidine 18.8 (11.6) mcg		
							midazolam 1.8 (0.6) mg		
Ayoglu [21]	IV	1 mcg/kg	10	patient-controlled sedation (5 mcg bolus dose; 10 min lockout)	no sedation	Patient-controlled sedation	dexmedetomidine 66.4 (3.7) mcg		
Dogan [13]	IV	0.6 mcg/kg	10	0.1–0.5 mcg•kg^− 1^•hr.^− 1^ (and topical analgesia or peribulbar block)	fentanyl 0.5 mcg/kg; and midazolam 20 mcg/kg then 5–15 mcg•kg^− 1^•hr.^− 1^ (and topical analgesia or peribulbar block)	RSS 3	Dexmedetomidine infusion	topical analgesia	8.54 mcg
								peribulbar block	8.92 mcg
							midazolam	topical analgesia	213.80 mcg
								peribulbar block	226.24 mcg
Erdurmus [14]	IV	1 mcg/kg	10	0.1–0.7 mcg/kg	saline	RSS 3	Unknown		
Ghodki [15]	IV	1 mcg/kg	10	–	saline	RSS 3	dexmedetomidine 65.4 (3.6) mcg		
Kermany [10]	IV	0.5 mcg/kg	10	0.2 mcg•kg^−1^•hr.^−1^	remifentanil 0.1 mcg/kg over 5 mins then 0.05 mcg•kg^− 1^•min^− 1^	BIS 70–80	Unknown		
Muttu [16]	IV	1 mcg/kg	20	0.05–0.7 mcg•kg^− 1^•hr.^− 1^	midazolam 50 mcg/kg then 2.5–35.0 mcg•kg^− 1^•hr.^− 1^	RSS 2	Unknown		
Na [17]	IV	–	NA	0.6 mcg•kg^− 1^•hr.^− 1^	propofol 2 mg•kg^− 1^•hr.^− 1^and alfentanil 20 mcg•kg^− 1^•hr.^− 1^	RSS 3	Unknown		
Park [22]	IV	0.5 mcg/kg	10	0.2 mcg•kg^−1^•hr.^− 1^	Remifentanil target effect site concentration 1 ng/ml	–	Unknown		
Virkilla [19]	IM	0.25 mcg/kg	NA	NA	placebo	45 mins before regional block	Unknown		
		0.5 mcg/kg							
		0.75 mcg/kg							
		1.0 mcg/kg							
		1.5 mcg/kg							
Virkilla [20]	IM	1.0 mcg/kg	NA	NA	midazolam 20 mcg/kg and placebo	45 mins before regional block	Unknown		
Yagan [18]	IV	0.5 mcg/kg	10	–	ketofol (200 mg propofol and 100 mg ketamine) 0.125 ml/kg	RSS 3	Unknown		

^a^*Route* route of study drug administration, data are, *IV* intravenous or, *IM*, intramuscular, ^b^Data are mcg/kg = micrograms per kilogram or mcg•kg^−1^•hr.^−1^ = micrograms per kilogram per hour; ^c^mins = minutes; ^d^Data are mcg•kg^− 1^•hr.^− 1^ = micrograms per kilogram per hour or mcg•kg^− 1^•min^− 1^ = micrograms per kilogram per minute; ^e^Data are total medication administered (standard deviation); ^f^*PRN* pro re nata, ^g^*RSS* Ramsay sedation scale score; ^h^*BIS* Bispectral index

Intravenous bolus doses of dexmedetomidine were given over 10–20 min and ranged from 0.5–1.0 mcg/kg [10, 12–16, 18, 21–23]. Continuous infusions of dexmedetomidine ranged from 0.05–0.7 mcg•kg^− 1^•hr.^− 1^ [10–14, 16, 17, 22–24]. Variability was also noted in the dosing of midazolam. Bolus doses of intravenous midazolam ranged from 20 to 50 mcg/kg [12, 16, 20]. Two studies titrated continuous infusions of midazolam at 25 mcg•kg^− 1^•hr.^− 1^ and 2.5–35.0 mcg•kg^− 1^•hr.^− 1^ to BIS > 85 and RSS 2, respectively [11, 16]. There was considerable variety in the total amount of dexmedetomidine and midazolam administered among studies that reported these values as shown in Table 2. Total doses of fentanyl, remifentanil, alfentanil, propofol, and ketamine were not reported in any of the studies. Although medication doses could be estimated from calculations using the average duration of surgery and patient weight, when reported, these calculations were not performed for this review.

### Hemodynamic and respiratory parameters

Cardiovascular depression was noted with dexmedetomidine as evidenced by statistically significant decreases in arterial pressure, heart rate, or both. Administration of dexmedetomidine by continuous infusion without initial bolus was not associated with statistically significant decreases in arterial pressure when compared to remifentanil, midazolam, and saline [11, 24]. However, when compared to propofol-alfentanil (combination of propofol and alfentanil), dexmedetomidine infusion titrated to RSS 3 without initial bolus dose led to statistically significant decreases in systolic blood pressure intraoperatively and postoperatively [17]. Additionally, hypotension was reported as an adverse event in 5 patients spanning 2 studies that used continuous infusions of dexmedetomidine at 0.4 mcg•kg^− 1^•hr.^− 1^and 0.6 mcg•kg^− 1^•hr.^− 1^ without initial boluses; arterial pressures that defined hypotension in these studies, however, were not specifically provided [17, 24]. Omitting a continuous infusion of dexmedetomidine was not associated with preserved arterial pressure or heart rate in any of the studies included in this review [15, 18–20]. Table 3 displays the effects of dexmedetomidine on arterial pressure and heart rate compared to other study medications and placebo.

**Table 3:** Effects of Dexmedetomidine on Arterial Pressure, Heart Rate, and Respiratory Parameters

Study	Dexmedetomidine		Other Treatment Group(s)	Arterial Pressure	Heart Rate	Respiratory Parameters
	Bolus	Continuous infusion				
Abdalla [23]	+	+	saline	Decreased SBP^a^ at 30 mins after starting infusion [SBP mean (SD^b^): 136.10 (15.24) vs 150.15 (21.73) mm Hg^c^, *P* = 0.043]	Decreased at 30 mins after starting infusion [HR^d^ mean (SD): 66.90 (5.42) vs 81.20 (7.83), *P* = 0.041] Decreased at 60 mins after starting infusion [HR mean (SD): 63.60 (4.55) vs 79.75 (7.83), *P* = 0.049]	–
Alhashemi [12]	+	+	midazolam	Decreased MAP^e^ [MAP (SE^f^): 86 (3) vs 102 (3) mm Hg, P < 0.05]	Decreased [HR (SE): 65 (2) vs 72 (2) bpm, *P* < 0.05]	No statistically significant differences reported
Altiparmak [24]	–	+	remifentanil	No statistically significant differences in MAP between groups	No statistically significant differences	Increased oxygen saturation at 1 min [mean SpO_2_^g^ (SD): 94.97 (2.0) vs 93.56 (2.69), *P* = 0.014], 5 mins [mean SpO_2_ (SD): 95.50 vs 92.84 (4.56), *P* = 0.001], 10 mins [mean SpO_2_ (SD): 95.52 (2.57) vs 92.65 (3.31), *P* = 0.000], 15 mins [96.02 (2.27) vs 93.34 (4.08), P = 0.001], and 20 mins [mean SpO_2_ (SD): 95.95 (2.43) vs 92.78 (4.15), P = 0.000] after inductionDecreased oxygen at 35 mins [mean SpO_2_ (SD): 93.14 (4.71) vs 97.0 (1.63), *P* = 0.002], 40 mins [mean SpO_2_ (SD): 94.29 (3.94) vs 97.08 (1.13), *P* = 0.027], and 45 mins [mean SpO_2_ (SD): 93.92 (4.17) vs 97.42 (1.51), *P* = 0.048] after induction
Apan [11]	–	+	midazolam and saline	No statistically significant differences in MAP between groups	Decreased 35–50 min after start of surgery and extending to 15 mins postoperatively [data values not reported, *P* < 0.05]	No statistically significant differences in respiratory rate or ETCO_2_^h^ values
Ayoglu [21]	+	+	no sedation	Decreased arterial pressure up to 30 mins after start of surgery [data values not reported, P < 0.05]	Decreased intraoperative mean HR up to 50 mins after start of surgery [data values not reported, *P* < 0.05]	No statistically significant differences in SpO_2_ or respiratory rate reported
Dogan [13]	+	+	midazolam-fentanyl with topical analgesia or peribulbar block	No statistically significant differences in SBP and DBP^i^ between groups	No statistically significant differences	No statistically significant differences in respiratory rate
Erdurmus [14]	+	+	saline	No statistically significant differences in SBP and DBP between groups	No statistically significant differences	No statistically significant differences in SpO_2_ between groups
Ghodki [15]	+	–	saline	Decreased MAP after premedication [mean MAP (SD): 86.88 (10.75) vs 99.10 (12.36), *P* = 0.005], intraoperatively [mean MAP (SD): 88.26 (11.03) vs 107.20 (7.90), *P* = 0.000], and postoperatively [mean MAP (SD): 81.02 (11.10) vs 90.03 (9.24), P = 0.008]	Decreased after premedication [mean HR (SD): 72.42 (13.25) vs 81.4 (7.36), P = 0.005], intraoperatively [mean HR (SD): 76.00 (14.21) vs 89.75 (9.21), P = 0.000], and postoperatively [mean HR (SD): 68.50 (11.54) vs 79.50 (15.68), *P* = 0.009]	No statistically significant differences in ETCO_2_ between groups
Kermany [10]	+	+	remifentanil	Decreased MAP postoperatively [mean MAP (SD): 76.3 (5.4) vs 85.2 (8.6), P = 0.01]	Decreased postoperatively [mean HR (SD): 65.4 (7.6) vs 72.1 (4.5), P = 0.009]	No statistically significant difference in SpO_2_ between groups
Muttu [16]	+	+	midazolam	No statistical analyses provided	No statistical analyses provided	No periods of desaturation in either group
Na [17]	–	+	propofol-alfentanil	Decreased SBP at surgery start [SBP (SD): 117 (16) vs 129 (19), P < 0.05], all intraoperative time points [SBP (SD): 117 (17) vs 129 (18), *P* < 0.05; 114 (15) vs 127 (18), P < 0.05; 112 (14) vs 126 (15), *P* < 0.05], and postoperatively [SBP (SD): 107 (15) vs 129 (18), P < 0.05]	No statistically significant differences between groups	No statistically significant difference in respiratory rate or SpO_2_ between groups
Park [22]	+	+	remifentanil	Decreased MAP [data values and P values not reported]	No statistically significant differences	Decreased ETCO_2_ and increased respiratory rate [data values not reported] No statistically significant differences in SpO_2_
Virkilla [19]	+	–	placebo	Decreased SBP in 1.5 mcg/kg group compared to placebo [data values not reported, P < 0.001]	Decreased in 1.5 mcg/kg group compared to placebo [data values not reported, *P* = 0.004]	No statistically significant changes in SpO_2_
Virkilla [20]	+	–	midazolam and placebo	Decreased SBP and DBP before periocular block compared to placebo [data values not reported, P < 0.001] Decreased SBP [data values not reported, P = 0.003] and DBP [data values not reported, P = 0.009] compared to midazolam before periocular block Decreased mean SBP and DBP compared to midazolam and placebo [data values not reported, *P* < 0.001] Decreased SBP postoperatively compared to midazolam and placebo [data values not reported, P < 0.001]	Decreased intraoperatively compared to placebo [data values not reported, P < 0.001] and midazolam [data values not reported, P = 0.01]Decreased postoperatively compared to placebo [data values not reported, P < 0.001]	Decreased SpO_2_ compared to midazolam and placebo before block [data values not reported, P < 0.001]
Yagan [18]	+	–	ketofol^j^	Decreased MAP after drug administration and all time points thereafter compared to baseline and ketofol [data values not reported, P < 0.05]	Decreased after drug administration and all time points thereafter compared to baseline [data values not reported, P < 0.05]	No statistically significant difference in respiratory rate

^a^*SBP* systolic blood pressure, ^b^*SD* standard deviation; ^c^*mm Hg* millimeters mercury, ^d^*HR* heart rate, ^e^*MAP* mean arterial pressure, ^f^*SE* standard error, ^g^*SpO*_*2*_ blood oxygen saturation, ^h^*ETCO*_*2*_ end-tidal carbon dioxide, ^i^*DBP* diastolic blood pressure, ^j^*ketofol* ketamine and propofol

Heart rate was preserved in 2 studies that did not administer a bolus dose of dexmedetomidine [17, 24]. However, one study comparing the effects of dexmedetomidine to midazolam or saline in 90 patients did not bolus dexmedetomidine, and reported statistically significant decreases in intraoperative and postoperative heart rates [11]. Bradycardia was reported as an adverse event in 5 studies and frequently required atropine treatment [15, 17, 19–21].

No statistically significant differences in oxygen saturation (SpO_2_), end-tidal carbon dioxide (ETCO_2_) values, or respiratory rates were reported in studies investigating the respiratory effects of dexmedetomidine compared to saline; no sedation; midazolam and fentanyl; propofol and alfentanil; and ketamine and propofol (ketofol) [10–15, 17–19, 21]. However, there were inconsistent results among studies investigating changes in ETCO_2_ and SpO_2_ when comparing dexmedetomidine to midazolam, placebo, and remifentanil [20, 22, 24]. One study demonstrated statistically significant decreases in oxygen saturation with IM dexmedetomidine 1 mcg/kg compared to midazolam 20 mcg/kg and placebo [20]. Better ventilation as evidenced by lower ETCO_2_ values and increased respiratory rates was noted in dexmedetomidine-treated patients compared to those receiving remifentanil titrated to a target effect site concentration of 1 ng/ml [22]. However, another study comparing dexmedetomidine to remifentanil infusion at 0.05 mcg•kg^− 1^•min^− 1^ reported statistically significant decreases in oxygen saturation in dexmedetomidine-treated patients 35–45 min after induction [24].

### Analgesia, sedation/cognitive dysfunction, and ponv

There were inconsistent results among studies that investigated pain perception during regional blocks and postoperative analgesia with dexmedetomidine compared to other study medications as shown in Table 4 [11, 13–16, 18, 20, 21, 24]. One study demonstrated better analgesia as measured by Verbal Pain Scale (VPS) score in patients receiving bolus doses of fentanyl 0.5 mcg/kg and midazolam 20 mcg/kg followed by midazolam infusion at 5–15 mcg•kg^− 1^•hr.^− 1^ compared to those receiving dexmedetomidine 0.6 mcg/kg followed by continuous dexmedetomidine infusion at 0.1–0.5 mcg•kg^− 1^•hr.^− 1^ titrated to RSS 3 [13].

**Table 4:** Effects of Dexmedetomidine on Analgesia, Sedation/Cognitive Dysfunction, Nausea/Vomiting, and PACU Discharge/Recovery Time

Study	Other Treatment Group(s)	Analgesia	Sedation or Cognitive Dysfunction	PONV^a^	PACU^b^ Discharge or Recovery Time
Abdalla [23]	saline	–	Increased sedation at 60 mins [mean BIS^c^ (SD^d^): 78.90 (4.99) vs 96.50 (2.09), *P* = 0.008] and 90 mins after starting infusion [93.00 (3.00) vs 96.35 (1.87), *P* = 0.047]	–	–
Alhashemi [12]	midazolam	–	–	–	Increased recovery time [median time required to achieve Aldrete 10 (IQR^e^) 45 (36–54) mins vs 21 (10–32) mins, *P* < 0.01]
Altiparmak [24]	remifentanil	Better analgesia at 10 mins [mean VRS^f^ (SD): 0.67 (0.69) vs 1.78 (1.68), *P* = 0.016], 15 mins [mean VRS (SD): 0.57 (0.59) vs 1.15 (1.0), P = 0.008], 20 mins [mean VRS (SD): 0.52 (0.55) vs 1.16 (1.04), *P* = 0.006], 25 mins [mean VRS (SD): 0.54 (0.60) vs 1.12 (1.02), *P* = 0.015], 30 mins [mean VRS (SD): 0.54 (0.56) vs 1.13 (1.02), *P* = 0.023], and 35 mins [mean VRS (SD): 0.42 (0.50) vs 1.09 (0.99), *P* = 0.024] after start of surgery	Increased sedation at 1 min [mean OAA/S^g^ (SD): 3.45 (0.74) vs 3.82 (0.93), *P* = 0.012] and 45 mins [mean OAA/S (SD): 4.00 (0.46) vs 3.30 (1.18), *P* = 0.003] following start of surgery Increased sedation at 1 min [mean BIS (SD): 86.1 (14.07) vs 91.95 (7.72), P = 0.024], 5 mins [mean BIS (SD): 79.92 (8.03) vs 88.76 (7.23), *P* = 0.000], 10 mins [mean BIS (SD): 76.92 (6.30) vs 84.35 (8.59), P = 0.000], 15 mins [mean BIS (SD): 76.77 (5.94) vs 83.39 (7.29), P = 0.000], 20 mins [mean BIS (SD): 76.67 (6.17) vs 82.76 (6.81), P = 0.000], and 45 mins [mean BIS (SD): 87.3 (8.4) vs 94.25 (1.75), *P* = 0.017] after start of surgery	–	–
Apan [11]	midazolam and saline	Better analgesia at 1 h postoperatively compared to midazolam [median VAS^h^ (range): 0 (0) vs 0 (13), *P* < 0.05] and saline [median VAS (range): 0 (0) vs 0 (12), P < 0.05]Better analgesia compared to midazolam at 2 h [median VAS (range): 0 (13) vs 8 (32), P < 0.05] and 3 h [median VAS (range): 5 (16) vs 13 (31), P < 0.05] postoperatively	Increased sedation compared to control group at 1 h [median 4-point sedation score (range): 2 (2) vs 1 (1), *P* < 0.05] and 3 h [median 4-point sedation score (range): 1 (1) vs 1 (1), P < 0.05] postoperatively	Not experienced by any patients	–
Ayoglu [21]	no sedation	Lower perception of pain during block [NRS^i^ (SD): 1.9 (0.5) vs 3.9 (0.6), P = 0.016]	Higher intraoperative RSS^j^ scores [data values not reported, P < 0.05]	–	No statistically significant differences in Aldrete scores
Dogan [13]	midazolam-fentanyl with topical analgesia or peribulbar block	Increased VPS [data values not reported, *P* = 0.001] Increased intraoperative analgesic requirement [data values not reported, P < 0.05]	No statistically significant differences in RSS scores between groups	Nausea and vomiting were observed in 1 patient ineach dexmedetomidine group	Increased time needed to achieve Aldrete 10 [data values not reported, P < 0.05]
Erdurmus [14]	saline	Decreased pain perception [mean VPS^k^ (SD): 1.23 (1.72) vs 3.64 (1.43), *P* < 0.001]	–	–	–
Ghodki [15]	saline	No statistically significant difference (NRS)	Increased [mean RSS (SD): 2.67 (0.48) vs 1.56 (0.44), *P* < 0.0001]	–	No statistically significant difference in time required to achieve Aldrete 10
Kermany [10]	remifentanil	–	Better cognitive outcomes postoperatively for patients younger than 65-years [postoperative MMSE^l^: 26.3 vs 25.5, *P* = 0.03] and older than 65-years [postoperative MMSE: 26.1 vs 24.1, *P* = 0.0001]	–	–
Muttu [16]	midazolam	No statistically significant difference [median VAS: 0.8 vs 1.1, *P* value not provided]	No persistent sedation in either group	–	–
Na [17]	propofol-alfentanil	–	–	–	–
Park [22]	remifentanil	–	No statistically significant differences between groups (BIS) Increased at 10 mins [data values not provided, *P* < 0.05]	–	Increased time needed to achieve RSS 2 [7.0–8.0 mins vs 1.9–1.7 mins, *P* value not provided]
Virkilla [19]	placebo	–	No statistically significant difference between doses (VAS)	–	–
Virkilla [20]	midazolam and placebo	No statistically significant differences in VAS associated with local anesthetic injection between treatment groups	No statistically significant differences compared to midazolam (VAS) Increased VAS compared to placebo [data not reported, *P* = 0.03] No statistically significant differences postoperatively between groups (VAS)	No statistically significant differences in nausea (VAS), and no patients vomited	–
Yagan [18]	ketofol^m^	No statistically significant difference in VAS scores during the block and 1, 2, and 4 h postoperatively	Decreased sedation following drug administration [data not reported, *P* < 0.001] and after block [data not reported, *P* < 0.017]	–	Increased recovery time [mean time to achieve Aldrete 9 (SD): 24.9 (4.5) vs 16.1 (2.1), P < 0.001]

^a^*PONV* postoperative nausea and vomiting, ^b^*PACU* post-anesthesia care unit, ^c^*BIS* bispectral index, ^d^*SD* standard deviation, ^e^*IQR* interquartile range, ^f^*VRS* verbal rating scale, ^g^*OAA/S* observer assessment of alertness/sedation, ^h^*VAS* visual analog scale, ^i^*NRS* numeric rating scale, ^j^*RSS* ramsay sedation scale, ^k^*VPS* verbal pain scale, ^l^*MMSE* mini-mental state examination, ^m^*ketofol* ketamine and propofol

Better cognitive outcomes as measured by performance on Mini-Mental State Examination (MMSE) were noted in a study comparing dexmedetomidine to remifentanil [10]. Increased intraoperative levels of sedation as measured by Observer Assessment of Alertness/Sedation Scale (OAA/S) and BIS were noted when dexmedetomidine was compared to remifentanil [24]. Increased sedation was also noted with dexmedetomidine compared to saline as measured by BIS and RSS [15, 23]. Increased postoperative sedation lasting up to 3 h postoperatively as measured by 4-point sedation score was reported in one study comparing dexmedetomidine to midazolam and saline [11].

Three studies commented on the incidence of PONV [11, 13, 20]. One study found no statistically significant difference in the subjective assessment of nausea in patients receiving IM dexmedetomidine compared to IM midazolam or placebo, but confidence intervals are unknown [20]. Another study comparing dexmedetomidine to midazolam and saline stated that PONV was not experienced by any patients, while another study reported nausea in 2 patients (2.5%) [11, 13].

### Pacu discharge and recovery times

Four studies investigated the time necessary to achieve a pre-determined target Aldrete score [12, 13, 15, 18]. One study noted that dexmedetomidine-treated patients required more time to achieve an Aldrete score of 10 when compared to midazolam-treated patients [median interquartile range (IQR): 45 (36–54) vs 21 (10–32) min, *P* < 0.01] [12]. Prolonged PACU discharge times were also noted in a study comparing dexmedetomidine to ketofol [mean time to achieve Aldrete score 9 (standard deviation (SD)): 24.9 (4.5) vs 16.1 (2.1) min, *P* < 0.001] [18]. These findings were not consistent with one study that utilized intravenous dexmedetomidine 1 mcg/kg followed by patient-controlled sedation with dexmedetomidine, which demonstrated no statistically significant differences in Aldrete scores at 30 min postoperatively compared to patients who received no sedation [21]. However, it is not clear from this study if the Aldrete scores achieved in 30 min were acceptable to discharge the patient, such as an Aldrete score 9 or 10 [21].

### Intraocular pressure

Seven studies in our review demonstrated significant reductions in IOP [13, 15, 18–21, 23]. One study reported a 3.8 mmHg decrease in IOP compared to control after a 1 mcg/kg bolus of dexmedetomidine [21]. Intramuscular dexmedetomidine was also shown to reduce IOP, and the greatest reductions were noted in patients who received 1 mcg/kg and 1.5 mcg/kg [mean IOP (SD): 6.8 (3.1) and 6.8 (3.0), respectively] [19]. Another study reported statistically significant decreases in IOP for patients who received dexmedetomidine [mean IOP (SD): 17.10 (1.92) to 13.81 (1.63), *P* < 0.0001] compared to those who received saline [mean IOP (SD):16.90 (4.11) to 15.41 (3.93), *P* > 0.05] [15]. Ketofol was also shown to reduce IOP at a rate similar to dexmedetomidine [18].

Preoperative decreases in IOP from dexmedetomidine were not sustained postoperatively. Dogan et al. identified no statistically significant differences in IOP between patients who received dexmedetomidine or the combination of fentanyl and midazolam at 1 h and 24 h following surgery [13]. These findings of non-sustained reductions in IOP are supported by another study comparing the effects of IM dexmedetomidine to IM placebo and IM midazolam, which reported similar mean IO*P* values in the operated eye in all groups 24 h after surgery [20].

### Patient and surgeon satisfaction

Compared to patients who received propofol-alfentanil, patients who received dexmedetomidine reported higher satisfaction scores [Iowa Satisfaction with Anesthesia Scale (ISAS) mean (SD): 50.3 (6.2) vs 42.7 (8.7), *P* < 0.001] [17]. Other studies corroborate this finding of higher patient satisfaction with dexmedetomidine compared to no sedation, saline, and midazolam [12, 14, 15, 21]. No statistically significant difference in patient satisfaction was noted when dexmedetomidine was compared to ketofol [mean 7-point Likert-like verbal rating scale (SD): 6.3 (0.5) vs 6.1 (0.7), *P* = 0.084] [18]. Furthermore, no statistically significant difference in patient satisfaction was noted when dexmedetomidine was compared to remifentanil [22].

Surgeon satisfaction was higher in 2 studies comparing dexmedetomidine to saline [14, 15]. There was no statistically significant difference in surgeon satisfaction when comparing dexmedetomidine to the combination of ketamine and propofol, or ketofol, [mean 7-point Likert-like verbal rating scale (SD): 6.4 (0.6) vs 6.2 (0.8), *P* = 0.067] or midazolam [median 7-point Likert-like verbal rating scale (IQR): 5 (4–6) vs 5 (4–6)] [12, 18]. Lower surgeon satisfaction was noted when dexmedetomidine was compared to remifentanil and midazolam-fentanyl [13, 22]. However, reasons for surgeon dissatisfaction were not specifically stated. Table 5 provides study details regarding patient and surgeon satisfaction.

**Table 5:** Effects of Dexmedetomidine on Patient and Surgeon Satisfaction

Study	Other Treatment Group(s)	Patient Satisfaction	Surgeon Satisfaction
Abdalla [23]	saline	–	–
Alhashemi [12]	midazolam	Increased satisfaction with sedation [median 7-point Likert-like verbal rating scale (IQR): 6 (6–7) vs 6 (5–7), *P* < 0.05]	No statistically significant difference (7-point Likert-like verbal rating scale)
Altiparmak [24]	remifentanil	–	–
Apan [11]	midazolam and saline	No statistical analyses of patient comments regarding satisfaction and effectiveness of sedation	No statistically significant difference (4-point scale)
Ayoglu [21]	no sedation	Increased NRS^a^ scores [data not reported, *P* = 0.001]	No statistically significant difference (NRS)
Dogan [13]	midazolam-fentanyl with topical analgesia or peribulbar block	Decreased [mean patient satisfaction on 5-point scale: 3.60 and 3.65 for patients receiving midazolam, fentanyl, and topical or peribulbar block, respectively; compared to 3.15 and 2.90 for patients receiving dexmedetomidine and topical or peribulbar block, respectively; *P* < 0.05]	Decreased [mean surgeon satisfaction on 5-point scale: 3.75 and 3.70 for patients receiving midazolam, fentanyl, and topical or peribulbar block, respectively; compared to 3.25 and 3.25 for patients receiving dexmedetomidine and topical or peribulbar block, respectively; P < 0.05]
Erdurmus [14]	saline	Increased score on 5-point scale [no data reported, *P* = 0.042]	Increased [mean surgeon satisfaction score on 5-point scale (SD^b^): 3.41 (0.80) vs 2.36 (1.26), *P* = 0.003]
Ghodki [15]	saline	Increased [mean patient satisfaction score on 10-point scale (range): 9 (8–10) vs 7 (5–8), *P* = 0.0001]	Increased [surgeon satisfaction score (excellent/good): 26/4 vs 13/17, P = 0.0001]
Kermany [10]	remifentanil	–	–
Muttu [16]	midazolam	–	No statistically significant difference on 4-point scale^d^
Na [17]	propofol-alfentanil	Increased [ISAS^c^ (SD): 50.3 (6.2) vs 42.7 (8.7), *P* < 0.001; and median ISAS (IQR): 50 (48–55) vs 45.0 (39–49)]	–
Park [22]	remifentanil	No statistically significant difference in 7-point Likert-like verbal rating scale	Decreased [mean 7-point Likert-like verbal rating scale (range): 6.05 (4–7) vs 6.35 (3–7), P < 0.05]
Virkilla [19]	placebo	–	–
Virkilla [20]	midazolam and placebo	–	–
Yagan [18]	ketofol^e^	No statistically significant difference in 7-point Likert-like verbal rating scale	No statistically significant difference in 7-point Likert-like verbal rating scale

^a^*NRS* numeric rating scale, ^b^*SD* standard deviation, ^c^*ISAS* Iowa Satisfaction with Anesthesia Scale, ^d^*4-point scale* poor, acceptable, good, or excellent, ^e^*ketofol* ketamine and propofol

## Discussion

There is noteworthy inconsistency in medication classes, doses, routes of administration, and outcomes analyzed in randomized clinical trials investigating the use of dexmedetomidine for sedation in outpatient cataract surgery. Although strict selection criteria could allow for meta-analysis, the number of relevant studies would be substantially reduced. In attempt to capture relevant outcomes for outpatient cataract surgery, we reviewed the impact of dexmedetomidine on hemodynamic parameters, respiratory parameters, pain, sedation, PONV, PACU discharge times, patient satisfaction, surgeon satisfaction, and IOP.

### Safety

#### Hemodynamic and respiratory parameters

Cardiovascular depression secondary to dexmedetomidine is consistent with the known effects of alpha-2 agonists and, particularly, the perioperative administration of dexmedetomidine [6, 7]. Our review demonstrates statistically significant decreases in arterial pressures and heart rates associated with dexmedetomidine in multiple studies utilizing various medication dosages and titration schemes. However, these decreases in arterial pressures and heart rates may not be clinically significant or negatively impact a patient’s perioperative course.

Although there appears to be some association between the bolus administration of dexmedetomidine and decreases in arterial pressures and heart rates, these findings are not supported by all studies. Specific data regarding arterial pressures, heart rates, and associated standard deviations are not retrievable from all publications. Many investigators graphically displayed these data without providing absolute values, thus precluding meta-analyses.

The ability to provide sedation and analgesia while also maintaining oxygenation and spontaneous ventilation is a commonly cited advantage of dexmedetomidine over alternative hypnotics, sedatives, and opioids [6, 7]. Multiple studies in this review demonstrated preserved oxygenation and ventilation [12, 17, 21, 22]. Statistically significant decreases in oxygen saturation in dexmedetomidine-treated patients were noted in two studies [20, 24]. However, the mean oxygen saturation did not fall below 93.14% (± 4.71%) in one study that reported the nadir [24]. Adverse respiratory events, such as the need for emergent intubation, were not reported in any study.

#### Post-operative cognitive dysfunction and sedation

Dexmedetomidine has been recommended as a safe therapy for reducing the incidence of post-operative delirium in the elderly after non-cardiac surgery in patients admitted to the ICU [25]. POCD and delirium have been associated with inflammatory mediators, which may be suppressed by dexmedetomidine [26, 27]. The incidence of POCD following cataract surgery has been reported at 4.4% and identified risk factors include advanced age and benzodiazepine pre-medication [28]. Overall, the commonest medications resulting in delirium (in the aging brain) include benzodiazepines, morphine, and anti-cholinergics [29]. Mansouri et al. identified no difference in the occurrence of POCD between midazolam and dexmedetomidine following cataract surgery under general anesthesia, although both were superior to placebo [30]. Unfortunately, the studies included in this review primarily focused on intraoperative levels of sedation and cognitive dysfunction, thus limiting our ability to draw conclusions regarding dexmedetomidine and postoperative neurocognitive disorders following cataract surgery.

The studies included in this review attempted to standardize the dosing of medications by targeting specific levels of intraoperative sedation. However, the methods used to assess sedation were variable. One study titrated dexmedetomidine and remifentanil to a BIS of 70–80 and described improved cognitive outcomes with dexmedetomidine as demonstrated by higher performance on MMSE [10]. Another study titrated dexmedetomidine and midazolam to a BIS greater than 85 and described persistent postoperative sedation on a 4-point scale [11]. Inconsistent study methods in assessing sedation preclude meta-analysis of these outcomes.

#### Nausea

Nausea is a known side effect of dexmedetomidine [6]. Only one study investigated the subjective assessment of nausea utilizing VAS and found no statistically significant difference between IM midazolam, IM dexmedetomidine, and IM placebo; however, the number of patients who experienced nausea was not provided and the confidence intervals for this result are unknown [20]. After combining the number of patients studied in 2 trials that defined nausea as an adverse event, only 2 patients (1.18%) reported nausea [11, 13].

### Efficacy

#### Analgesia

Pain is one of the most powerful predictors of return to normal activity after surgery [31]. Although pain after cataract surgery is often considered minimal, Porela-Tiihonen reported that 34% of patients had some pain and 9% had more than moderate pain (VAS > 4) in the first few hours after cataract surgery [32].

Better analgesia was noted in patients treated with dexmedetomidine compared to remifentanil, midazolam, saline, and placebo in several studies [11, 14, 21, 24]. These findings are consistent with the known pharmacodynamic properties of dexmedetomidine. However, multiple studies reported no statistically significant difference in analgesia when comparing dexmedetomidine to saline, placebo, midazolam, and ketofol [15, 16, 18, 20]. Peribulbar blocks can provide complete analgesia for cataract surgery, which may limit the ability to identify statistically significant differences in analgesia between dexmedetomidine and other medications or placebo in patients following cataract surgery. Most studies did not investigate analgesia beyond 1 h postoperatively, at which time the peribulbar block may start to dissipate and potentially reveal differences in analgesia between study medications or placebo. Only one study compared dexmedetomidine to the common pairing of midazolam and fentanyl, which exhibited better analgesia [13].

#### Intraocular pressure

Dexmedetomidine reduces intraocular pressure by its central action [33]. Kim et al. reported that dexmedetomidine attenuated the increases in IOP associated with steep Trendelenburg positioning during laparoscopic radical prostatectomy [34]. Reduced eye pressure may improve surgical operating conditions and decrease the risk for elevated IOP following cataract surgery [35].

Multiple studies in our review demonstrated significant reductions in IOP [13, 15, 18–21, 23]. However, these decreases in IOP were not sustained postoperatively. These findings may be related to the relatively short half-life of dexmedetomidine, the short duration of surgery during which dexmedetomidine was infused, or both.

#### Recovery time

Our review demonstrates a tendency for prolonged recovery in patients who received dexmedetomidine, which may limit its utility in the ambulatory surgery setting. When compared to ketofol or midazolam, patients treated with dexmedetomidine required longer times to achieve an Aldrete score of 9 or 10, respectively [12, 18]. In these studies demonstrating prolonged recovery, medications were titrated to RSS 3, which was a common sedation target of other studies included in this review. These findings may be the clinical manifestations of differences in context-sensitive half-times between medications. Overall, PACU discharge and recovery times were infrequently reported by investigators.

#### Satisfaction

Patients who received dexmedetomidine generally reported higher satisfaction scores [12, 14, 15, 17, 21]. Each of the articles included in this review studied patients undergoing monitored anesthesia care, a form of anesthesia in which patient cooperation with the surgeon is critical. Therefore, despite unknown validity and reliability, we considered surgeon satisfaction to be a key component in comparing sedative agents. Surgeons most frequently reported no statistically significant difference in satisfaction associated with the administration of dexmedetomidine compared to other agents [11, 12, 16, 18, 21]. However, lower satisfaction was noted by surgeons when dexmedetomidine was compared to remifentanil [22]. The combination of midazolam and fentanyl was the only study medication group other than saline that was associated with higher patient and surgeon satisfaction scores [13]. Specific reasons for patient or surgeon dissatisfaction were not provided, thus limiting our ability to further analyze these outcomes.

### Limitations

This systematic review is limited by inconsistent study medications, doses, routes of administration, and primary study outcomes of randomized clinical trials. Additionally, the publications included in this review demonstrate variability in the quality of randomization, double-blinding, and reporting of withdrawals and dropouts. These limitations preclude meta-analyses.

## Conclusion

Dexmedetomidine offers several advantages over traditional sedatives, hypnotics, and opioids in adult patients undergoing outpatient cataract surgery, such as better analgesia, preserved respiratory function, reduced intra-ocular pressure, and higher patient satisfaction. However, dexmedetomidine is frequently associated with cardiovascular depression, particularly when administered in bolus doses ranging from 0.5–1.0 mcg/kg, and may delay PACU discharge. The advantages and disadvantages of perioperative dexmedetomidine for outpatient cataract surgery should be considered on a patient-by-patient basis.

## Data Availability

The datasets supporting the conclusions of this article are included within the article.

## Abbreviations

ASA: American Society of Anesthesiologists’
BIS: Bispectral index
DBP: Diastolic blood pressure
ETCO_2_: End-tidal carbon dioxide
HR: Heart rate
hr.: hour
IM: Intramuscular
IOP: Intraocular pressure
IQR: Interquartile range
ISAS: Iowa Satisfaction with Anesthesia Scale
IV: Intravenous
ketofol: ketamine-propofol
kg: kilogram
MAP: Mean arterial pressure
mcg: microgram
mcg/kg: micrograms per kilogram
mcg•kg^− 1^•hr.^− 1^: micrograms per kilogram per hour
mcg•kg^− 1^•min^− 1^: micrograms per kilogram per minute
mg: milligrams
min: minute
mm Hg: millimeters of mercury
MMSE: Mini-mental state examination
n: number
NRS: Numeric rating scale
OAA/S: Observer assessment of alertness/sedation scale
PACU: Post-anesthesia care unit
POCD: Postoperative cognitive dysfunction
PONV: Post-operative nausea and vomiting
PRN: Pro re nata
RSS: Ramsay sedation scale
SBP: Systolic blood pressure
SD: Standard deviation
SE: Standard error
SpO_2_: Oxygen saturation
VAS: Visual analog scale
VPS: Verbal pain scale
VRS: Verbal rating scale
